# Prostatic Response to Supranutritional Selenium Supplementation: Comparison of the Target Tissue Potency of Selenomethionine *vs.* Selenium-Yeast on Markers of Prostatic Homeostasis

**DOI:** 10.3390/nu4111650

**Published:** 2012-11-06

**Authors:** David J. Waters, Shuren Shen, Seema S. Kengeri, Emily C. Chiang, Gerald F. Combs, J. Steven Morris, David G. Bostwick

**Affiliations:** 1 Department of Veterinary Clinical Sciences, Purdue University, West Lafayette, IN 47907, USA; 2 The Center on Aging and the Life Course, Purdue University, West Lafayette, IN 47907, USA; 3 Gerald P. Murphy Cancer Foundation, West Lafayette, IN 47906, USA; Email: seleniumhealth@gpmcf.org (S.S.); seema@gpmcf.org (S.S.K.); chiang1@purdue.edu (E.C.C.); 4 USDA-ARS Grand Forks Human Nutrition Research Center, Grand Forks, ND 58203, USA; Email: gerald.combs@ars.usda.gov; 5 University of Missouri-Columbia Research Reactor Center, Columbia, MO 65211, USA; Email: morrisj@missouri.edu; 6 Harry S. Truman Memorial Veterans Hospital, Columbia, MO 65201, USA; 7 Bostwick Laboratories, Glen Allen, VA 23060, USA; Email: dbostwick@bostwicklaboratories.com

**Keywords:** prostate cancer, cancer prevention, carcinogenesis, SELECT, animal models

## Abstract

Prostate cancer is the product of dysregulated homeostasis within the aging prostate. Supplementation with selenium in the form of selenized yeast (Se-yeast) significantly reduced prostate cancer incidence in the Nutritional Prevention of Cancer Trial. Conversely, the Selenium and Vitamin E Cancer Prevention Trial (SELECT) showed no such cancer-protective advantage using selenomethionine (SeMet). The possibility that SeMet and Se-yeast are not equipotent in promoting homeostasis and cancer risk reduction in the aging prostate has not been adequately investigated; no direct comparison has ever been reported in man or animals. Here, we analyzed data on prostatic responses to SeMet or Se-yeast from a controlled feeding trial of 49 elderly beagle dogs—the only non-human species to frequently develop prostate cancer during aging—randomized to one of five groups: control; low-dose SeMet, low-dose Se-yeast (3 μg/kg); high-dose SeMet, high-dose Se-yeast (6 μg/kg). After seven months of supplementation, we found no significant selenium form-dependent differences in toenail or intraprostatic selenium concentration. Next, we determined whether SeMet or Se-yeast acts with different potency on six markers of prostatic homeostasis that likely contribute to prostate cancer risk reduction—intraprostatic dihydrotestosterone (DHT), testosterone (T), DHT:T, and epithelial cell DNA damage, proliferation, and apoptosis. By analyzing dogs supplemented with SeMet or Se-yeast that achieved equivalent intraprostatic selenium concentration after supplementation, we showed no significant differences in potency of either selenium form on any of the six parameters over three different ranges of target tissue selenium concentration. Our findings, which represent the first direct comparison of SeMet and Se-yeast on a suite of readouts in the aging prostate that reflect flux through multiple gene networks, do not further support the notion that the null results of SELECT are attributable to differences in prostatic consequences achievable through daily supplementation with SeMet, rather than Se-yeast.

## 1. Introduction

**Figure 1:**
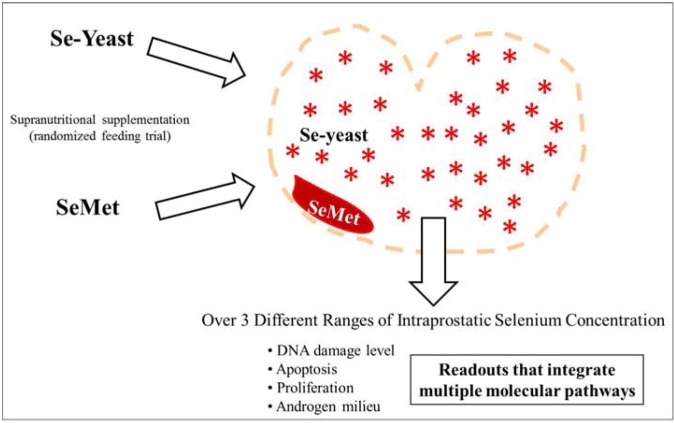
*Attempting to explain the disappointing results of selenomethionine supplementation in SELECT: Does selenium-yeast have superior activity in prostatic tissue?* Strong prostate cancer risk reduction with selenized yeast (Se-yeast) was reported in men in the Nutritional Prevention of Cancer (NPC) Trial. In contrast, men in SELECT received no such cancer protective advantage from supplementation with selenomethionine (SeMet). In an attempt to explain these disparate results and the apparent superiority of Se-yeast, researchers are wondering whether these two forms of selenium are equally capable of promoting prostatic homeostasis and lowering cancer risk. In the randomized dietary supplementation paradigm in dogs reported here, Se-yeast was not superior to SeMet in boosting systemic or target tissue selenium concentrations; equal oral dosing with Se-yeast or SeMet yielded comparable post-supplementation selenium concentrations measured in toenails and within prostatic tissue (See Results section of this manuscript). Thus, the idea that form-dependent differences in resultant intraprostatic selenium concentration might explain the disappointing results of SELECT is not supported here. In this paper, we sought to validate an alternative explanation: Could the remarkable prostate cancer risk reduction seen in the NPC Trial with Se-yeast, but not seen with SeMet in SELECT, reflect that supplementation with Se-yeast works better within the prostate to promote homeostasis and reduce prostate cancer risk (*Se-yeast depicted in this cartoon as stars throughout the prostate*), whereas SeMet supplementation is less active within the prostate in terms of homeostasis and cancer risk reduction (*SeMet depicted in this cartoon as biologically sequestered, possessing a limited working effect within the prostate*)? The particular molecular target for selenium’s anticancer effect within the prostate has not been elucidated. Further, the effects of these two forms of selenium on this target may be dose-dependent. Conceding then that the optimal concentration within the prostate to activate the key molecular pathway is unknown, we probed the biological consequences of reaching three different ranges of intraprostatic selenium concentration. Specifically, we determined whether individuals receiving Se-yeast or SeMet that achieve a particular level of intraprostatic selenium post-supplementation would show significant differences in six measures of homeostasis within the prostate. The readouts we measured—DNA damage, cell proliferation, apoptosis, testosterone (T), dihydrotestosterone (DHT) and DHT:T—capture flux through multiple signaling pathways and reflect processes that likely influence cancer risk within the prostate. The cartoon predicts that when Se-yeast supplemented and SeMet supplemented individuals that reach equivalent intraprostatic selenium concentrations are compared, the prostates of individuals receiving Se-yeast will have significantly different readouts of DNA damage, proliferation, apoptosis, or hormonal milieu compared to SeMet supplemented prostates. Alternatively, if no differences are found in the readouts between the two forms across three different ranges of intraprostatic selenium concentration, these observations would lend no further support for the scenario set forth in the cartoon—that Se-yeast has superior activity in prostatic tissue (See text for Results and Discussion).

Prostate cancer is the product of dysregulated homeostasis within the aging prostate. Daily selenium supplementation in the form of selenized yeast (Se-yeast) significantly reduced prostate cancer incidence in men in the Nutritional Prevention of Cancer (NPC) Trial [1]. Conversely, the Selenium and vitamin E Cancer Prevention Trial (SELECT) showed no such cancer-protective advantage using selenomethionine (SeMet) [2]. It has been postulated that the null results of SELECT may reflect that the form of selenium used in the trial, selenomethionine, was ill-advised [3,4,5]. Although SeMet is the major component of Se-yeast [6,7], what remains shrouded in uncertainty is whether SeMet or Se-yeast can elicit superior prostatic responses (Figure 1). This is because data from direct *in vivo* comparison of the effects of these two selenium forms on prostatic homeostasis and cancer risk are lacking. Pin-pointing the form-dependent, prostatic consequences of selenium supplementation might meaningfully aid in placing the results of SELECT in proper context, offering valuable insights into guiding future work on the potential use of selenium supplementation as a cancer preventive strategy.

Using the dog prostate model, we previously showed a non-linear, U-shaped dose response relationship between toenail selenium status and prostatic DNA damage in a randomized feeding trial in dogs, producing a broad range of toenail selenium concentrations mimicking that of U.S. men [8]. Recently, a meta-analysis of the dose-response between selenium and prostate cancer risk reduction in men confirmed a U-shaped relationship between toenail selenium and risk for prostate cancer, but conceded that further research on the cancer-protective potency of different selenium forms was needed [9]. In our studies, dogs were randomized to receive two different doses of organic selenium supplementation in the form of either SeMet or Se-yeast, but the results we have reported to date have never explored the extent of form-dependent differences. 

If the critical target of selenium for cancer risk reduction were known, then the research imperative would logically be focused on comparing the consequences of SeMet and Se-yeast supplementation at the specific prostate tissue concentration that optimizes target activity. Because this knowledge of target is currently beyond our grasp, we pursued an alternative investigative approach that acknowledges our current state of understanding and incorporates two assumptions: (1) we do not know the critical molecular target of selenium within the prostate; and it follows that (2) we do not know the optimal intraprostatic selenium concentration for cancer risk reduction. We reasoned that analyzing prostatic tissue from SeMet or Se-yeast supplemented dogs that achieved equivalent intraprostatic selenium concentration after supplementation could provide a valuable test to determine whether either of these two forms of selenium exert superior activity on key markers of prostatic homeostasis that integrate multiple molecular pathways. 

Here, using our valuable preclinical cohort of dogs, we report the first direct comparison which interrogates whether SeMet or Se-yeast differentially influences steady-state intraprostatic selenium concentration. Further, we conduct a comparative analysis of *target tissue potency* by evaluating whether SeMet or Se-yeast shows differential effects within the prostate on markers of intraprostatic androgens, DNA damage, proliferation, and apoptosis—a collection of readouts in a chemopreventive setting that reflect alterations in multiple prostatic gene networks and processes that likely influence cancer risk reduction [10,11,12,13,14]. Because prostatic response to cancer-fighting nutrients is likely dose-dependent, we report the impact of SeMet and Se-yeast on these integrated markers over three different ranges of target tissue selenium concentration. 

## 2. Experimental Section

Methods and observations from this experimental cohort have been previously reported [8,15,16]. Relevant details pertaining to study design, assessment of selenium status and biological effects within the prostate, and data analysis are described briefly here.

### 2.1. Study Design

In a randomized controlled feeding trial, 49 elderly beagle dogs, physiologically equivalent to 62–69-year-old men [17], received nutritionally adequate or supranutritional levels of selenium for 7 months to produce a range of steady-state selenium levels that mimicked those seen in healthy adult U.S. men. Dogs were randomly assigned to a control group (*n* =10 dogs) or four daily oral treatment groups: 3 or 6 μg Se/kg/day in the form of SeMet (L-selenomethionine, Solgar Vitamin and Herb, Leonia, NJ, USA) or Se-yeast (SelenoExcell^®^, Cypress Systems, Fresno, CA, USA). The selenium in the Se-yeast product is mostly (~75%) SeMet [7]. At baseline, all dogs had nutritionally adequate selenium status confirmed by plasma selenium concentration. After seven months, all dogs were euthanized in accordance with guidelines set forth by the American Veterinary Medical Association Panel on Euthanasia. All aspects of this experimental protocol were approved by the Purdue University Animal Care and Use Committee.

### 2.2. Assessment of Selenium Concentration in Toenails and Prostate

Toenail clippings were collected at baseline (*t* = 0) and immediately after euthanasia (*t* = 7 months). Snap frozen prostate tissue was collected from dogs immediately after euthanasia. Selenium was determined in toenails and prostate tissues by instrumental neutron activation analysis at the University of Missouri-Columbia Research Reactor Center (MURR), Columbia, MO using methods previously described [18]. 

### 2.3. Assessment of Intraprostatic Testosterone (T) and Dihydrotestosterone (DHT) Concentration by RIA

Snap frozen prostate tissue was homogenized for 1 min with a Polytron homogenizer in cold PBS (pH 7.5) in a ratio of 1 mg tissue/10 µL PBS. The homogenate of the prostate was extracted with hexane and ethyl acetate. Intraprostatic concentration of T and DHT were measured using commercially available RIA kits (Active Testosterone RIA DSL-4000; Active Dihydrotestosterone RIA DSL-9600; Diagnostic Systems Laboratories, Inc., Webster, TX, USA). 

### 2.4. Assessment of Prostatic DNA Damage

Fresh prostate tissue (50 to 80 mg) was collected at necropsy to prepare prostate cell suspensions [8]. Cytospin preparations showed that >90% of cells had epithelial cell morphology; mean percentage cell viability estimated by trypan blue exclusion was 80%. Histopathologic evaluation of formalin-fixed, step-sectioned prostate tissue sections revealed no foci of carcinoma. The extent of DNA damage in prostate cells, which is an index of oxidative stress and other genotoxic influences within the prostate, was measured by single cell gel electrophoresis (alkaline Comet assay) [19]. Each cell was visually scored on a 0 to 4 scale using a method described by Collins [20]. The extent of DNA damage within the prostate was expressed as the percentage of cells with extensive damage (sum of type 3 and type 4 cells).

### 2.5. Assessment of Proliferative Index and Apoptosis within the Prostate

Five micron-thick tissue sections were prepared from each formalin-fixed prostate. The avidin-biotin-complex technique (Vector Laboratories, Burlingame, CA, USA) was used. Monoclonal antibodies were used to determine proliferative index (anti-MIB-1, Vector Laboratories) [21]. Each section was scanned at low power to identify the region with the highest percentage of cells with MIB-1 positive nuclear staining. Proliferative index was expressed as the percentage of MIB-1 positive cells determined in a 200× field within this hot spot.

A modification of the TUNEL method was used to determine the frequency of apoptosis [22]. For each dog, the number of prostatic epithelial cells with positive nuclear staining was counted in randomly selected, non-contiguous, 200× microscopic fields. Immunopositive stromal cells, inflammatory cells, or epithelial cells that were shed into the acinar lumen were not counted. Microscopic fields that contained areas that displayed intense inflammation were not scored. The median number of apoptotic prostatic epithelial cells per 200× field was represented as an apoptotic index. 

### 2.6. Statistical Analysis

Mann-Whitney U test was used to test for differences in the form-dependent effects of SeMet or Se-yeast supplementation on median toenail selenium concentration and median intraprostatic selenium concentration. The two forms of selenium were compared at each dose level. Spearman correlation coefficients were calculated separately for SeMet and for Se-yeast supplementation to determine the extent to which toenail and intraprostatic selenium concentrations were correlated. Form-dependent effects of selenium supplementation on six prostatic parameters were evaluated—intraprostatic DHT, T, DHT:T ratio; prostatic DNA damage, proliferative index, and apoptotic index. To determine the extent of selenium form-dependent differences in potency, all selenium-supplemented dogs were combined then subdivided into tertiles based upon intraprostatic selenium concentration achieved after supplementation. Then, within each tertile of intraprostatic selenium concentration, Mann-Whitney U test was used to compare median values for the six prostatic parameters in SeMet *versus* Se-yeast supplemented individuals. All data analyses were done using standard statistical software [SPSS (Version 18.0, Chicago, IL, USA) and SAS System (Version 9.2, SAS Institute, Cary, NC, USA, 1999)]. 

## 3. Results

### 3.1. Absence of Form-Dependent Effects of Selenium Supplementation on Systemic Selenium Status

**Figure 2:**
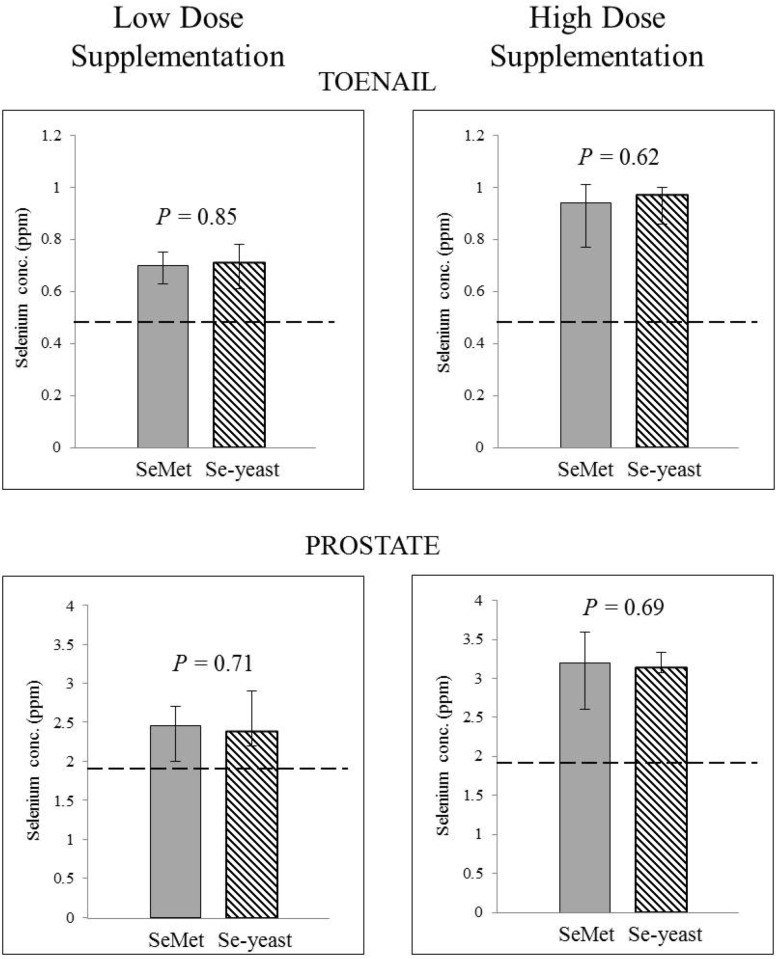
Form-dependent effects of selenium supplementation on selenium status as measured by toenail and intraprostatic selenium concentration. Each bar presents median ± inter-quartile range. Interquartile range indicates the difference between the first and third quartiles. *P* values represent Mann-Whitney U test comparing low-dose selenomethionine (SeMet) with low-dose selenized yeast (Se-yeast), and comparing high-dose SeMet with high-dose Se-yeast. The dashed line indicates the median value of toenail or intraprostatic selenium concentration in unsupplemented dogs, which differs significantly from the median toenail and intraprostatic selenium concentration of each of the four selenium supplemented groups (Mann-Whitney U Test, *p* < 0.05). ppm: parts per million.

**Figure 3:**
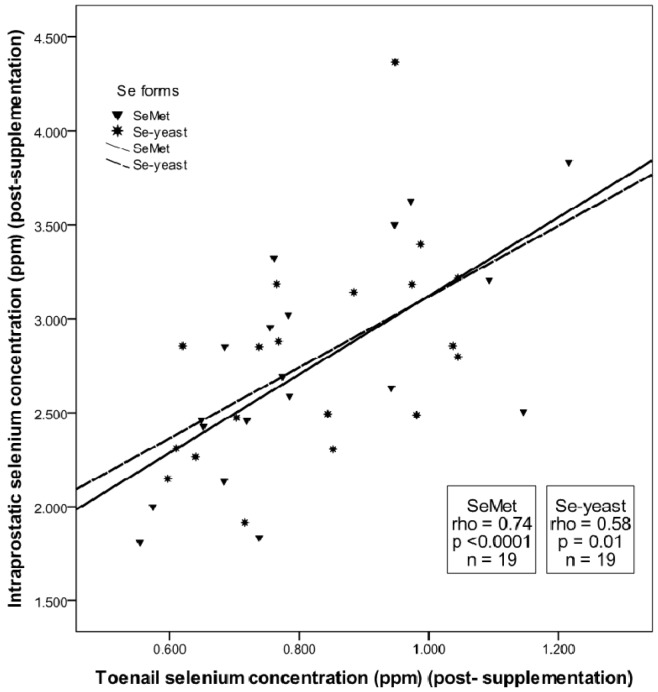
Correlation between toenail and intraprostatic selenium concentration after seven months of supplementation with selenomethionine (SeMet) or selenized yeast (Se-yeast).

No significant between-group differences in toenail selenium concentration were evident at baseline (*t* = 0). Supplementation significantly increased toenail selenium concentration above pre-supplementation levels in each of the selenium groups (Mann Whitney U Test, *p* < 0.05). But there was no significant form-dependent difference in the magnitude of increase in toenail selenium over the seven month supplementation period in the SeMet and Se-yeast groups (low dose: median change = 0.02 ppm *versus* 0.05 ppm, respectively; *p* = 0.41; high dose: median change = 0.30 ppm *versus* 0.30 ppm, respectively; *p* = 0.65). Toenail selenium concentration after seven months of supplementation with either form of selenium were comparable at both low and high dose (Figure 2). Toenail selenium concentration after seven months of supplementation with either form of selenium had a significant, linear correlation with intraprostatic selenium concentration at seven months (SeMet: Spearman rho = 0.74, *p* < 0.0001; Se-yeast: Spearman rho = 0.58, *p* = 0.01) (Figure 3).

### 3.2. Absence of Form-Dependent Effects of Selenium Supplementation on Intraprostatic Selenium Concentration

After seven months of supplementation, there was no significant difference between intraprostatic selenium concentration in the low-dose SeMet and low-dose Se-yeast groups (median 2.46 ppm *versus* 2.39 ppm, respectively; *p* = 0.71). Likewise with high-dose supplementation, there was no significant difference between intraprostatic selenium concentration in dogs supplemented with SeMet or Se-yeast (median 3.20 ppm *versus* 3.14 ppm, respectively; *p* = 0.69) (Figure 2). With these results, we could extend our analysis beyond a comparison of the two forms based upon oral dose to a more coveted biological comparison: In an experimental dietary supplementation paradigm achieving equivalent post-supplementation intraprostatic selenium status, would SeMet and Se-yeast exert equipotent biological effects within the prostate?

### 3.3. Prostatic Response to SeMet *vs.* Se-yeast Supplementation: Analysis of Target Tissue Potency Using Markers of Prostatic Homeostasis

To determine the extent of form-dependent differences in the prostatic response to selenium, we evaluated the effects of selenium supplementation by tertiles of intraprostatic selenium concentration achieved after supplementation. Within each tertile, SeMet and Se-yeast supplemented dogs were compared for differences in six prostatic parameters—intraprostatic DHT, T, DHT:T, DNA damage, proliferative index, and apoptotic index. This enabled us in a chemopreventive setting to interrogate over a range of post-supplementation intraprostatic selenium concentration whether SeMet or Se-yeast could elicit more potent effects on indicators of prostatic homeostasis that likely influence cancer risk. Table 1 summarizes the results of this potency analysis. Across three different ranges of intraprostatic selenium status and six prostatic parameters, there were no significant form-dependent differences in the potency of SeMet and Se-yeast within the prostate (Table 1).

**Table 1:** Prostatic response to selenomethionine (SeMet) *vs.* selenium-yeast (Se-yeast): analysis of target tissue potency using markers of prostatic homeostasis.

		Tertiles of Intraprostatic Selenium Concentration ^†^		
Prostatic markers *		Lowest	Middle	Highest
DHT (ng/g tissue)	SeMet (*n*)	6.6 (7)	5.7 (6)	6.1 (6)
	Se-yeast (*n*)	5.9 (6)	6.2 (7)	6.3 (6)
	*p*	*0.89*	*0.48*	*0.69*
T (ng/g tissue)	SeMet (*n*)	2.1 (7)	2.6 (6)	3.2 (6)
	Se-yeast (*n*)	2.7 (6)	2.7 (7)	3.1 (6)
	*p*	*0.67*	*0.57*	*0.87*
DHT:T	SeMet (*n*)	2.7 (7)	2.0 (6)	2.4 (6)
	Se-yeast (*n*)	2.5 (6)	2.9 (7)	1.9 (6)
	*p*	*0.57*	*0.15*	*0.52*
DNA damage (%)	SeMet (*n*)	55 (7)	60 (6)	58 (6)
	Se-yeast (*n*)	52 (6)	50 (7)	52 (6)
	*p*	*0.31*	*0.22*	*0.16*
Apoptotic index (%)	SeMet (*n*)	2.0 (7)	1.5 (6)	2.5 (6)
	Se-yeast (*n*)	4.0 (6)	2.0 (7)	1.5 (6)
	*p*	*0.93*	*0.61*	*0.75*
Proliferative index (%)	SeMet (*n*)	1.1 (6)	0.6 (5)	0.4 (6)
	Se-yeast (*n*)	0.3 (5)	0.6 (5)	1.3 (4)
	*p*	*0.14*	*0.91*	*0.58*

Values are median; *n* is number of dogs in each treatment group; DHT: dihydrotestosterone; T: testosterone; *p* values represent Mann-Whitney U test comparing the effect of SeMet and Se-yeast on markers within each tertile of intraprostatic selenium concentration; * prostatic marker values (median) in the unsupplemented control group are 4.6 (DHT), 2.1 (T), 2.2 (DHT:T), 79 (DNA damage), 0.5 (apoptotic index) and 0.9 (proliferative index); median values for DNA damage and apoptosis in controls differed significantly (*p* < 0.05) from selenium-supplemented dogs; **^†^** range for tertiles of intraprostatic selenium concentration achieved after 7 months supplementation were as follows: lowest (1.84–2.47 ppm), middle (2.49–2.96 ppm), and highest (3.00–4.36 ppm); for comparison, in two studies of men receiving short-term (2 to 6 weeks) supplementation with 200 μg of selenium prior to radical prostatectomy, intraprostatic selenium concentration ranged from 1.2 to 4.1 ppm [23] and 1.5 to 4.3 ppm [24].

## 4. Discussion

Great hope for developing selenium as a practical approach for prostate cancer risk reduction was invested in SELECT, the largest-ever prostate cancer prevention trial [25]. However, null findings dashed earlier optimism raised by the NPC Trial that daily selenium supplementation might lead to significant cancer risk reduction. Speculating on an explanation for these disappointing results, SELECT might have suffered from using SeMet, rather than Se-yeast employed in the NPC Trial [3,4,5]. It was reasonable to believe that previously unforeseen differences in prostatic activity between SeMet and Se-yeast might be at the root of the discrepancy. Such a difference in cancer-protective potential might emanate from differences in their biodistribution [26,27] or activity within the prostate (Figure 1). We reasoned that a detailed, form-specific analysis of our selenium work in elderly dogs—the only non-human species to frequently develop prostate cancer during aging [28,29]—would enable direct comparison of these two supplements, guiding a deeper search for potential form-dependent differences in prostatic distribution and activity of selenium *in vivo*.

First, we probed for form-dependent differences in systemic selenium status after supplementation. We found equivalent increases in toenail selenium concentration after SeMet or Se-yeast supplementation at both low and high doses. Likewise, we found no form-dependent differences in intraprostatic selenium concentration. Thus, we found no evidence that supplementation with SeMet is less capable than Se-yeast to boost intraprostatic selenium level. Whether or not supplementation with SeMet or Se-yeast differentially alters the amount of particular selenium species within the prostate, such as methylselenol or selenoproteins [30,31], could not be determined in our study, because only total selenium concentration was measured. Whether or not host factors, such as age or obesity [32], impact the systemic and intraprostatic concentrations of selenium achievable after supplementation with different selenium forms could not be rigorously interrogated in our dogs due to small sample size, but deserves further evaluation.

But this result—SeMet and Se-yeast supplementation in an equivalent dose yield equivalent systemic and intraprostatic selenium concentrations—positioned us to probe the important question: Does supplementation with these two forms exert differential biological effects within the prostate *in a context of equivalent selenium status*? Pursuant to that question, we conducted the first direct comparison of the potency of SeMet and Se-yeast on a suite of prostatic readouts that reflect alterations in multiple gene networks. To search for form-dependent differences in the capacity of selenium to influence prostatic responses, we conducted a tertile analysis of all selenium-supplemented dogs based on final intraprostatic selenium concentration. This potency analysis revealed that when dogs receiving SeMet or Se-yeast were standardized on the basis of their intraprostatic selenium concentration achieved after supplementation, there were no significant differences in the effect of either selenium form on epithelial cell DNA damage, proliferation, apoptosis, or intraprostatic androgen milieu. Across three different ranges of selenium status and six parameters, supplementation with SeMet or Se-yeast yielded equivalent outcomes. These results have strong translational implications. Our findings, which represent the first direct comparison of SeMet and Se-yeast on biological markers in the aging prostate that reflect flux through multiple gene networks, do not support the idea illustrated in Figure 1 that the null results of SELECT are attributable to a superior response of the aging prostate to daily supplementation with Se-yeast, rather than SeMet. Instead, based on the significant anticancer efficacy of Se-yeast in men with low baseline selenium in the NPC Trial [33] together with the equipotent effects of the two forms of selenium reported here, we speculate that subgroup analysis of prostate cancer risk in SELECT will show that men with low baseline selenium concentration can achieve prostate cancer risk reduction from supplementation in the form of SeMet.

Lippman and colleagues [2] pointed out that it was impossible to know whether Se-yeast would have been more active than SeMet had it been used in SELECT. No inferences could be made because SELECT did not test different formulations of selenium. Unfortunately, direct comparison of the effects of SeMet and Se-yeast on the prostate is not available from any human studies. Investigators have reported dose-dependent increases in intraprostatic selenium concentration in men after short-duration supplementation (2 to 6 weeks) with SeMet [23] or Se-yeast [24,34], but other prostatic responses to supplementation were not reported. Making hard conclusions based on between-study comparisons is difficult, owing to differences in dose and duration of supplementation, study subjects, and assays used to measure selenium. The power of the comparative analysis of SeMet and Se-yeast presented here is that potency was evaluated in a randomized, preclinical setting in which biological effects within the prostate could be evaluated in individuals having *equivalent intraprostatic selenium status*. We believe this experimental approach affords the kind of hard-to-achieve estimate of form-dependent activity that is needed to reach sounder conclusions regarding potential differences in the effects of SeMet and Se-yeast on prostatic homeostasis and cancer risk reduction. The notion that a retreat to animal studies might yield a more complete understanding of disappointing human clinical trials has precedence in the CARET and ATBC lung cancer prevention trials. Subsequent studies in ferrets triggered an illuminating contextual re-think, revealing the dangerous combination of current smoking and high-dose beta-carotene, which ultimately led to increasing confidence that the 10% increase in lung cancer observed in β-carotene-supplemented smokers could be reconciled as *an expected*, *rather than an unexpected outcome* [35].

In summary, the null results of SELECT strengthened enthusiasm that there may be important form-dependent differences in selenium action within the prostate. The results of our novel potency analysis communicated here do not further support that notion. By guiding more informed speculations and provoking new questions, new data will continue to shape the ongoing dialogue about SELECT and its implications for defining the efficacy of selenium supplementation as a cancer-preventive strategy. By faithfully reporting our observations, we fortify that intellectual debate. 
